# Knowledge, Attitude, and Perception Regarding the Human Papillomavirus (HPV) Vaccine Among Parents at Al-Madinah Al-Munawwar: A Cross-Sectional Study

**DOI:** 10.7759/cureus.65850

**Published:** 2024-07-31

**Authors:** Faris Altom, Nussaiba Y Khawaji, Mona M Almalki, Wejdan A Almohammadi, Heyam S Al-Enezi, Shayma Y Al-Khalil

**Affiliations:** 1 Basic Sciences, Al Rayan National College of Medicine, Madinah, SAU; 2 Medicine and Surgery, Al Rayan National College of Medicine, Madinah, SAU

**Keywords:** hpv vaccine, knowledge, attitude, saudi parents, human papillomavirus

## Abstract

Background: Human papillomavirus (HPV) is the leading cause of cervical cancer in reproductive-age Saudi women. Parents' understanding and attitude regarding HPV vaccination in young girls are vital to preventing cervical cancer.

Objective: This study aims to assess the knowledge, attitudes, and perceptions of parents in Al-Madinah Al-Munawwara towards the HPV vaccine and identify factors influencing their decision to vaccinate their children.

Methods: A cross-sectional survey was conducted among 500 parents in Al-Madinah Al-Munawwara. A structured questionnaire was used to collect data on demographics, knowledge about HPV and the HPV vaccine, attitudes towards vaccination, and perceptions of vaccine safety and efficacy. Data were analyzed using Statistical Product and Service Solutions (SPSS, version 21; IBM SPSS Statistics for Windows, Armonk, NY).

Results: Parents have 57.6% knowledge about HPV, 69.2% perceive it as dangerous, and 29.8% know its link to cervical cancer. Physicians are the primary source, and 81.2% believe the vaccine protects HPV. Key predictors of vaccine acceptance included higher educational levels, awareness of HPV-related health risks, and recommendations from healthcare professionals.

Conclusion: The study reveals a lack of knowledge about HPV infection and vaccines among Saudi Arabian parents, with only 7.2% having vaccinated their children, emphasizing the need for education and screening programs.

## Introduction

Human papillomavirus (HPV) is a prevalent sexually transmitted infection (STI) known for causing various epithelial lesions and is one of the most widespread STIs affecting the genital tract. With over 100 distinct types, HPV can lead to genital warts, abnormal cervical cells, and even cervical cancer [1]. The global prevalence of HPV infection in women without cervical abnormalities is around 11-12%, with higher rates observed in regions, such as sub-Saharan Africa, Eastern Europe, and Latin America.

HPV infection is strongly associated with various cancers, including cervical, penile, vulvar, vaginal, anal, and oropharyngeal cancers. Estimates indicate an annual incidence of genital warts at 0.1-0.2%, with a peak occurring during teenage and young adult ages [2]. HPV-related cervical cancer, which develops in the cells lining the lower uterus, is the fourth most common cancer globally. In 2020, the World Health Organization reported over 600,000 new cases globally, resulting in 342,000 fatalities. Notably, it ranks as the eighth most common cancer among Saudi Arabian women aged 15-40, with approximately 358 new cases diagnosed annually and 179 resulting in death [3].

The HPV vaccine serves as the primary line of defense against HPV-related illnesses and malignancies. Introduced in stages starting with the bivalent (2vHPV) vaccination for girls in 2006, followed by approval of the quadrivalent (4vHPV) vaccine for boys in 2009, and the availability of the 9-valent HPV vaccine for both genders in 2014, vaccination is recommended ideally between ages 9 and 14, before sexual debut [4,5]. Research has focused particularly on teenagers, especially girls aged 15-25, emphasizing the vaccine's efficacy against various HPV types [6].

The Centers for Disease Control recommends routine HPV vaccinations typically starting at age 11, extending to age 26, to mitigate the risk of HPV-associated cancers [7]. Despite the vaccine's proven benefits, achieving high vaccination rates globally remains challenging due to diverse socio-economic, cultural, and access-related factors [8-10]. Parental acceptability plays a crucial role in enhancing vaccination coverage, influenced by perceptions of vaccine safety and efficacy for children and adolescents [11]. Recent studies have underscored the significant benefits of early HPV vaccination in reducing cervical cancer risk among vaccinated women, reinforcing the importance of vaccination programs [12].

In Saudi Arabia, however, awareness and acceptance of the HPV vaccine remain low [13]. This study seeks to assess parents' knowledge, attitudes, and perceptions towards the HPV vaccine in Al-Madinah Al-Munawwara, identifying factors that influence their decision-making regarding the vaccination of their children.

## Materials and methods

Study design, setting, and population

An analytical cross-sectional questionnaire-based study was carried out among Saudi parents from October 2023 to March 2024. We used a pre-designed questionnaire to gather information about parents' awareness and acceptance of the HPV vaccine in Al-Medina. The inclusion criteria for this study include parents of girls in primary schools aged 9-11 years who live in Al-Medina. We excluded parents with children over 11 years old and parents who do not reside in Al-Medina.

Sample size

EPI Info™ (CDC, Atlanta, GA) calculated a sample size of 500 based on the total population in the western region, which was 85,230, at a 95% confidence interval (CI) and a 5% margin of error. We collected data from a convenient sample of 500 Saudi parents using an online questionnaire.

Sampling technique

Participants for the survey were selected using a convenient sampling method. We targeted parents of girls in primary schools aged 9-11 years who live in Al-Madinah Al-Munawwara. The survey was distributed both online and in person to ensure a diverse and representative sample. The online surveys were distributed via school email lists and parent groups on social media platforms, while the in-person surveys were conducted during parent-teacher meetings and school events.

Data collection

To gather information, we sent paper surveys to parents of girls in primary schools aged 9-11 years old. We divided the survey into three parts, gender, marital status, level of schooling, job, and monthly income during the first part. The second part employed nine questions to assess the subjects' understanding of HPV and the HPV vaccine. The last question asked if the person needed to learn more about the human papillomavirus. The third part of the study looked at how people felt about the HPV vaccine, whether they were willing to vaccinate their daughters, why they did not want to vaccinate their daughters, how much they knew about their children's previous HPV vaccinations, and whether they had access to their children's vaccination records.

Pre-testing

The pre-testing process involved a sample size of 50 parents, representative of the study's target population. The questionnaire was initially distributed to this group to assess clarity, reliability, and overall effectiveness. Based on the feedback received, several adjustments were made to the questionnaire, including rephrasing ambiguous questions, adjusting the format for better readability, and ensuring that the content was culturally appropriate.

Study tools

The questionnaire used in this study was developed based on existing literature and expert consultation to ensure its relevance and comprehensiveness. It was pre-tested with a small sample to ensure clarity and reliability. The questionnaire consisted of multiple-choice and Likert-scale questions to capture a wide range of responses.

Assessment of the questionnaire

The knowledge assessment section assessed the participants' knowledge levels based on their responses. We awarded one point for each correct answer, resulting in a possible total score of nine points. We classified participants with an accuracy rate of at least 80% (at least seven correct answers) as having a high level of knowledge and those with scores below 80% as having a low level of knowledge.

Data analysis

We statistically analyzed the data using Statistical Product and Service Solutions (SPSS, version 26; IBM Corp., Armonk, NY). Participants with an accuracy rate of at least 80% were classified as having a high level of knowledge, while those with a score below 80% were classified as having a low level. The chi-square test and frequency were performed to examine the demographic differences between participants who knew about HPV, its relation with cervical cancer, availability of vaccine for the prevention of cervical cancer, and acceptance of the HPV vaccine. A P-value below 0.05 was considered to be statistically significant.

Ethical considerations

We asked each participant to sign an ethical consent form in writing. The Al Rayyan Medical Colleges (AMC) Ethical Committee developed and authorized the informed ethical consent form (Grant No. HA-03-M-122-081).

## Results

In our study, 38% (195) of the parents were aged between 36 and 45 years; 65% (326) were females, and 35% (174) were males, as shown in Table 1.

**Table 1:** Distribution of studied participants according to their demographics (N = 500) Data represented as N (number of participants = 500) and percentage (%).

Variable	N (%)
Age (years)	
15-25	70 (14%)
26-35	111 (22.2%)
36-45	195 (38.2%)
>45	124 (24.8%)
Gender	
Female	326 (65.2%)
Male	174 (34.8%)

A total of 57.6% of parents had previous knowledge about HPV, 69.2% thought that HPV is very dangerous, and only 29.8% knew the harmful association between HPV infection and the occurrence of cervical cancer (Table 2). The most common source of their knowledge was the internet (35%). A total of 50.6% thought that the vaccine has side effects, and 81.2% reported that it prevents HPV infection. The majority (69.6%) reported the need for more education about HPV.

**Table 2:** Distribution of studied participants according to their response to knowledge items regarding HPV and HPV vaccine (N = 500) Data represented as N (number of participants = 500) and percentage (%).

Variable	N (Percentage)
Has knowledge about Human papillomavirus (HPV)	
No	212 (42.4%)
Yes	288 (57.6%)
HPV is very dangerous	
No	77 (15.4%)
Yes	346 (69.2%)
Maybe	77 (15.4%)
Harm that is associated with the infection with Human papillomavirus (HPV)	
Cancer of the breast	54 (10.8%)
Cancer of the anus, penis, vagina, vulva, and the back of the throat	202 (40.4%)
Cancer of the cervix	149 (29.8%)
Negatively affects fertility	52 (10.4%)
Unknown	43 (8.6%)
Has knowledge about the Human papillomavirus (HPV) vaccine	
No	148 (29.6%
Yes	352 (70.4%)
Source of knowledge about the HPV vaccine (N = 113)	
Physicians	34 (30%)
Internet	40 (35%)
Family and friends	20 (18%)
Social media	19 (17%)
Vaccine has any side effects	
No	123 (24.6%)
Yes	253 (50.6%)
Maybe	124 (24.8%)
Vaccine prevents the infection with HPV	
No	47 (9.4%)
Yes	406 (81.2%)
Maybe	47 (9.4%)
Vaccine reduces the severity of symptoms after getting infected	
No	101 (20.2%)
Yes	299 (59.8%)
Maybe	100 (20%)
Target group for the vaccine	
Unmarried females 9 to 25 years old	99 (19.8%)
Married females 9 to 25 years old	141 (28.2%)
Before marriage	206 (41.2%)
Don't know	54 (10.8%)
Facility that can introduce the HPV vaccine	
Schools	98 (19.6%)
Primary health care	187 (37.4%)
Hospitals	165 (33%)
Don't know	50 (10%)
Need for more education about HPV	
No	152 (30.4%)
Yes	348 (69.6%)

**Figure 1:**
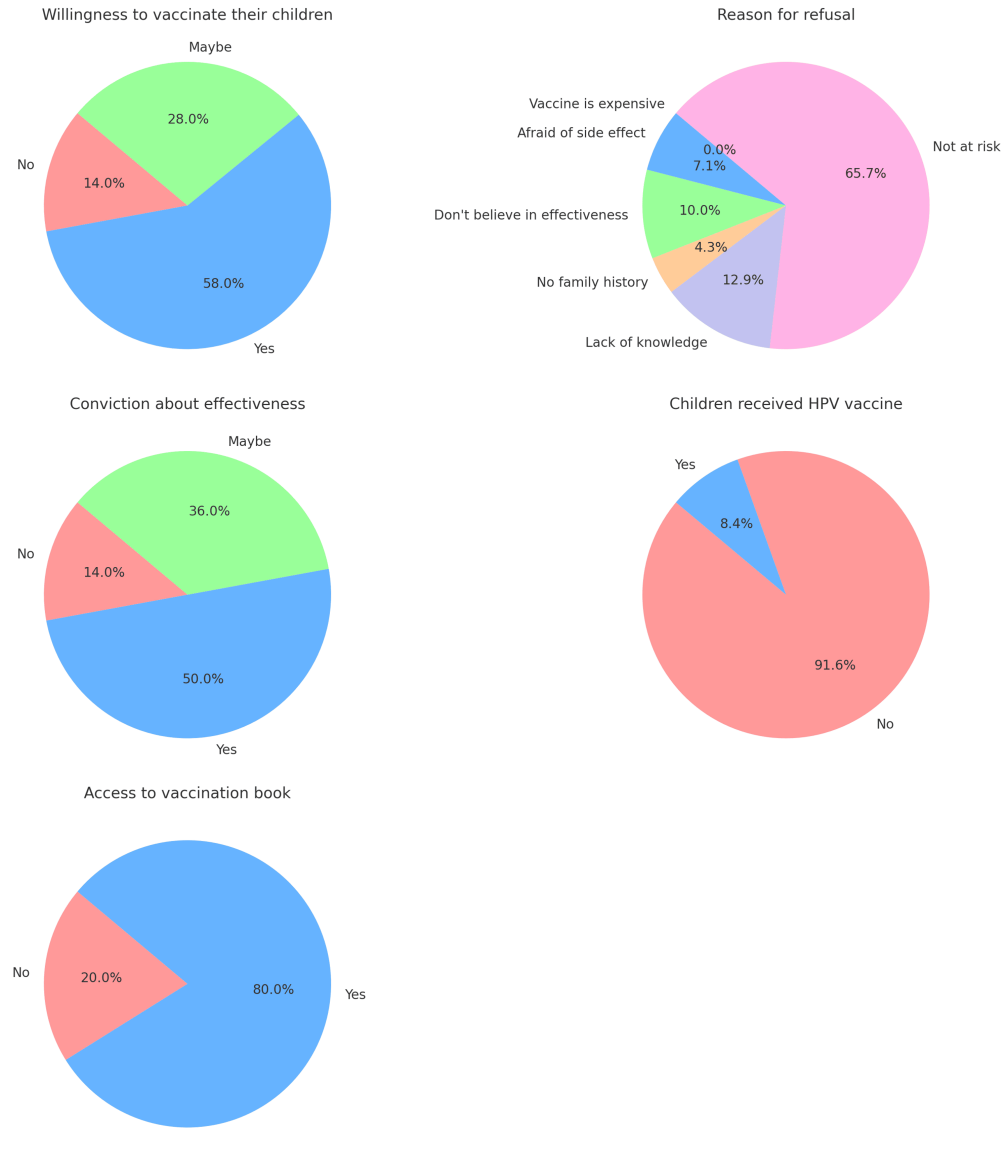
Attitude and willingness to vaccinate their children with the HPV vaccine and influencing factors by study group

Figure 1 shows that 58% are willing to vaccinate their children, 28% are uncertain, and 14% are unwilling. The primary reason for refusal is the belief that you are not at risk (65.7%). While 50% believe in the vaccine's effectiveness, only 8.4% of children have received it. Additionally, 80% of parents have access to a vaccination book.

The percentage of individuals with a high level of knowledge was significantly higher among employed parents, those who obtained their knowledge from physicians, those who had access to the content of their children's vaccination book, and those who were convinced of the vaccine's effectiveness for their children (P = 0.080, 0.050, 0.080, 0.070, 0.050), as shown in Table 3.

**Table 3:** Relationship between the knowledge level and participants' demographics (N = 500) HPV, human papillomavirus; SR, Saudi Riyals; *Statistically significant (P < 0.05). All values presented as numbers (N) and percentages (%).

Variable	Knowledge level			
	Poor	Good	Chi-square value	P-value
	N (Percentage)	N (Percentage)		
Age (years)				
15-25	70 (14%)	0 (0%)	1.075	0.3
26-35	110 (22%)	10 (2%)		
36-45	180 (36%)	20 (4%)		
>45	140 (28%)	10 (2%)		
Gender				
Female	260 (52%)	3 (0.6%)	0.904	0.2
Male	170 (34%)	67 (13.4%)		
Marital status				
Widow	20 (4%)	5 (1%)	0.562	0.4
Married	340 (68%)	40 (8%)		
Divorced	70 (14%)	25 (5%)		
Education level				
Intermediate school	30 (6%)	10 (2%)	0.755	0.25
Secondary school	80 (16%)	10 (2%)		
Diploma	70 (14%)	30 (6%)		
University	240 (48%)	20 (4%)		
Postgraduate	80 (16%)	10 (2%)		
Employment status				
Employed	90 (18%)	110 (22%)	7.867	0.080*
Unemployed	290 (58%)	10 (2%)		
Student	20 (4%)	0 (0%)		
Monthly income (SR)				
<5000	50 (10%)	20 (4%)	1.043	0.28
5000-10,000	120 (24%)	100 (20%)		
10,000-20,000	170 (34%)	80 (16%)		
>20,000	50 (10%)	50 (10%)		
None	110 (22%)	20 (4%)		
Source of knowledge about the HPV vaccine				
Physicians	70 (14%)	130 (26%)	5.049	0.050*
Internet	80 (16%)	120 (24%)		
Family and friends	40 (8%)	80 (16%)		
Social media	50 (10%)	60 (12%)		
Have access to the content of their children's vaccination book				
No	210 (42%)	40 (8%)	0.06	0.080*
Yes	170 (34%)	80 (16%)		
Have a conviction about the effectiveness of the vaccine for their children				
No	120 (24%)	80 (16%)	4.0432	0.050*
Yes	80 (16%)	130 (26%)		
Maybe	110 (22%)	20 (4%)		

## Discussion

In Saudi Arabia, raising awareness of cervical cancer is essential to forming good habits and taking preventative action. The vaccine has been available since 2006, and HPV is responsible for 99% of occurrences of cervical cancer [14]. Studies on obesity, diabetes, and breast cancer are already being conducted; however, very few look at Saudis' awareness of the HPV vaccine and cervical cancer [15].

The present study, which covered the Al-Madinah Al-Munawwara, revealed that participants had little understanding of HPV. According to the survey, only 29.8% of parents recognized that the damage linked with HPV infection is cancer of the cervix; 57.6% of them had prior information about HPV, and 69.2% considered that HPV is quite harmful. The low understanding of HPV and its vaccination could be attributed to the fact that the community in the Kingdom of Saudi Arabia typically only knows about the common mandatory vaccinations for their children, rather than a new and uncommon vaccination such as HPV. From our perspective, another reason for this lack of knowledge could be the insufficient health education provided by the primary healthcare system.

King Faisal University's 2014 survey found that a significant number of the 181 Saudi medical students were not aware of the early signs, symptoms, and risk factors associated with cervical cancer. These findings are consistent with the studies cited earlier. The average accuracy rates varied between 43.7% and 55% [16]. Furthermore, a study demonstrated that Polish students aged 17-26 exhibited a deficient comprehension of cervical cancer and did not see HPV infection as the primary determinant [17]. A recent survey done among healthcare practitioners in Greece has unveiled a notable dearth of knowledge concerning HPV. Only 30% of individuals were aware of the significant impact of HPV on cancer development [18]. A recent survey in Thailand revealed a significant lack of knowledge and understanding about HPV [19].

The current study's participants (57.6%) were aware of the HPV vaccine; 50.6% believed that the vaccine had various negative effects, and their physicians were the most prevalent source of information (29.8%). The results align with a 2014 study in Saudi Arabia, where 67% of participants lacked knowledge about HPV vaccination [19].

The majority of the current research subjects (69.6%) indicated that they required health education regarding HPV. This is a highly amenable matter, as it indicates the public's willingness to enhance their awareness. Consequently, it is imperative to provide community education.

Furthermore, this study revealed that 58.0% of parents expressed a willingness to immunize their children with the HPV vaccination, while 28.0% said that they were uncertain about vaccinating them. The primary obstacle for individuals who declined immunization was their perception of being immune to infection (64.2%). Over 50% of parents believed that the vaccination was effective for their children, whereas only 13% had actually immunized their children against HPV. Out of the total, 80.0% were able to view the contents of the children's vaccination book. The findings align with a previous study from Riyadh in 2016, where a mere 37% of participants declined the vaccine [20]. Over 55% of participants in a recent nationwide research survey in Saudi Arabia expressed a strong desire to receive the HPV vaccination if it became available to them. Additionally, 73% of respondents stated that they would actively endorse the HPV vaccine to others. Furthermore, more than 50% of medical students in Jeddah expressed a strong interest in acquiring the HPV vaccination [21].

Working parents, parents who got health information from their doctors, parents who had already vaccinated their daughters, parents who could read their daughters' vaccination book, and parents who believed the vaccine would work for their kids all knew a lot about HPV vaccination. It was not surprising that this occurred, as working parents may have learned about the new trend of getting the HPV vaccine from their coworkers, particularly since middle schools now mandate it for all female students. Furthermore, parents who received medical information from doctors knew more accurate facts about the HPV vaccine than their peers who obtained it from a non-specialized source. Parents who wanted to confirm their children's vaccination history could learn more about the HPV vaccine, a new addition to their vaccination schedule. Explaining the purpose and health benefits of a new drug or vaccine can easily convince someone to take it. Because of this, parents who knew a lot about the vaccine were more sure that it worked.

Researchers found that parents who were married, had a high level of education, and received health information from their doctors were more likely to vaccinate their children. This conclusion makes sense since parents who are married and have a lot of education would likely know more about and easily understand the health benefits of this vaccine, which would protect their children from a very dangerous disease such as cervical cancer. This is why they would be more open to this treatment than other people. Doctors can also encourage their patients and clients to receive the vaccine by emphasizing its benefits and using evidence-based medicine.

At the end of the study, 54% of parents who knew a lot about the HPV vaccine were ready to give it to their kids (P = 0.050). This result indicates the need for more health education campaigns throughout the Kingdom of Saudi Arabia, not just in Madinah, to increase public awareness of this significant issue. All basic healthcare facilities in the region already offer the HPV vaccine, which women can use to prevent cervical cancer. We need to educate everyone about this and spread the word.

Study limitations

Firstly, participants self-reported the number of vaccinated children, which may introduce measurement bias due to recall accuracy and educational background. Secondly, the online data collection process might affect data reliability. Additionally, comparing the study results with HPV statistics from only a few countries other than Saudi Arabia limits the scope. A broader comparison with multiple countries could have better highlighted whether the lack of awareness is a significant issue or a common trend.

Despite these challenges, the study reveals Saudi parents' open attitudes towards the HPV vaccine, as well as their limited knowledge and action regarding this crucial vaccine. This research lays the groundwork for further studies in this field and may encourage health organizations to provide community education classes on cervical cancer, emphasizing the importance of early detection and vaccination.

## Conclusions

The study highlights the importance of parental vaccination and the need for improved health education. There is a significant gap in knowledge about HPV and its vaccine. Educational campaigns targeting parents and healthcare providers can help reduce the prevalence of HPV-related diseases, including cervical cancer, in Saudi Arabia. Schools, primary healthcare centers, and hospitals can serve as effective platforms for these campaigns. Future research should explore the long-term impact of educational interventions and the effectiveness of communication strategies in increasing vaccine uptake. This study underscores the need for a concerted effort to improve HPV-related health education.
